# *Cryptosporidium* Oocyst Contamination in Drinking Water: A Case Study in Italy

**DOI:** 10.3390/ijerph16112055

**Published:** 2019-06-10

**Authors:** Cristina Pignata, Silvia Bonetta, Sara Bonetta, Simone M. Cacciò, Anna R. Sannella, Giorgio Gilli, Elisabetta Carraro

**Affiliations:** 1 Department of Public Health and Pediatrics, University of Torino, Via Santena 5bis, 10126 Torino, Italy; cristina.pignata@unito.it (C.P.); sara.bonetta@unito.it (S.B.); giorgio.gilli@unito.it (G.G.); elisabetta.carraro@unito.it (E.C.); 2 Istituto Superiore di Sanità, Viale Regina Elena 299, 00161 Roma, Italy; simone.caccio@iss.it (S.M.C.); annarosa.sannella@iss.it (A.R.S.)

**Keywords:** *Cryptosporidium* spp., waterborne disease, rural areas, RT-PCR, genotyping

## Abstract

The aim of this study was to evaluate the occurrence of *Cryptosporidium* oocysts in a drinking water treatment plant (DWTP) located in a rural area of northern Italy. Influent and effluent samples were collected at the DWTP over three years (2013–2016). In parallel, tap water samples from a public drinking fountain were collected as well. All samples were analyzed for the presence of *Cryptosporidium* spp. oocysts by a common method based on an immunomagnetic separation (IMS)/immunofluorescence assay (IFA), complemented by 4,6-diamidino-2-phenylindole (DAPI) staining. A reverse transcriptase-PCR (RT-PCR) protocol was added to evaluate oocyst viability. The results highlighted a high variability of oocyst concentrations across all samples (mean 4.3 ± 5.8/100 L) and a high variability in the percentage of DAPI-positive specimens (mean 48.2% ± 40.3%). Conversely, RT-PCR did not reveal the presence of viable *C. parvum* and *C. hominis* oocysts. A nested PCR targeting *Cryptosporidium* 18S ribosomal DNA, carried out in two water samples, confirmed the presence of a *Cryptosporidium* genotype associated with wild animals in the river and in tap water. The results obtained underline the vulnerability of the investigated surface water to *Cryptosporidium* spp. contamination. Although the recovered *Cryptosporidium* genotype is not a human pathogen, its presence demonstrates the existence of a potential pathogen *Cryptosporidium* spp. contamination risk. Moreover, these results underline the importance of also considering unconventional (not bacterial) biological contaminations (protozoa) in water resources in rural areas, including those of developed countries.

## 1. Introduction

Despite the best available technologies applied in drinking water production and the control and surveillance systems put in place, waterborne diseases are still a public health problem even in industrialized countries.

In the United States, 928 waterborne outbreaks were reported from 1971 to 2014. Over forty years, various aetiological agents have caused repeated contaminations. Bacteria, viruses, and unidentified agents have caused most of these epidemics, but outbreaks by parasites such as *Cryptosporidium* spp. and *Giardia intestinalis* have also consistently occurred throughout this period [1].

A similar situation has also occurred in Europe, where in 2017, seven member states of the European Union reported 27 waterborne outbreaks caused by different microorganisms, including calicivirus (norovirus), *Campylobacter*, *Cryptosporidium*, hepatitis virus, ‘Viruses other than hepatitis A virus and Calicivirus, Shiga toxin-producing *E. coli*, parasites (other than *Cryptosporidium* and *Trichinella*) and unknown agents [2]. Between January 2011 and December 2016, 381 waterborne outbreaks due to parasitic protozoa (e.g., *Giardia* spp., *Cryptosporidium* spp.) were reported worldwide. Forty-nine percent of the events occurred in New Zealand, 41% in North America and 9% in Europe [3].

*Cryptosporidium* spp. display a global distribution, and a number of species are recognized as human pathogens, although most cases are due to *C. hominis* and *C. parvum*. These microorganisms were considered responsible for most of the waterborne outbreaks associated with *Cryptosporidium* typed to date, except for a few other species as confirmed by the outbreak in the UK caused by *C. cuniculus* [4]. Pathogenic *Cryptosporidium* gives rise to self-limiting gastrointestinal diseases that are difficult to cure with current drugs and potentially lethal in immunocompromised subjects [5,6].

The first documented epidemic due to the consumption of water contaminated by *Cryptosporidium* spp. was reported in the United States in 1985, and the largest outbreak caused by *Cryptosporidium* spp. occurred in the city of Milwaukee in 1993. Based on epidemiological studies conducted in that area, it was possible to verify that the number of subjects who had manifested the symptoms of gastroenteritis between March and April 1993 amounted to approximately 403,000 people. Four thousand and four hundred individuals were admitted to various hospital facilities, and at least 69, most of whom were immunocompromised, eventually died. The origin of this event was attributed to the inefficiency of one of the two drinking water treatment plants catching water from nearby Lake Michigan. In the period immediately preceding the outbreak, strong spring thunderstorms had increased the lake turbidity, causing an increase in the passage of particulates and reasonably also of oocysts of *Cryptosporidium* spp. throughout the plant [7].

Over the past decade, several outbreaks of cryptosporidiosis have been reported in many EU countries in association with the consumption of contaminated drinking water, recreational waters (especially swimming pools), food consumption and contact with animals [8,9,10].

In the United Kingdom, cases occurred more frequently in spring, mostly due to *C. parvum*, and were presumably generated by increased exposure to oocysts from young animals, in conjunction with the season of births [11]. In recent years, the phenomenon has lessened due to greater veterinary checks and, above all, to the quality improvement of water for human consumption [12]. At the same time, however, there has been an increase in contamination during the summer–autumn period caused mainly by *C. hominis*; this increased contamination is probably linked to an increase in exposure of the subjects to contaminated recreational water [13].

In 2010–2011, the two largest epidemics ever reported in Europe were recorded in Sweden. Approximately 47,000 people from two different cities (Östersund and Skellefteå) were involved. *C. hominis* caused both outbreaks, and the consumption of water from a contaminated supply source was identified as the cause of these two outbreaks [14,15].

In August 2013, in the German city of Halle, 167 people, mostly children, showed the classic symptoms of cryptosporidiosis. The outbreak was caused by the consumption of water contaminated by wastewater following the flooding of the Saale River. Analysis of environmental samples showed a high number of oocysts (up to 592 oocysts/100 L) of *C. hominis* [10].

In Italy, the only documented outbreak linked to the consumption of water contaminated by *Cryptosporidium* spp. occurred in 1995 in the Emilia Romagna Region, in a drug rehabilitation community (1,731 people). Two hundred ninety-four people manifested acute diarrhea, with a higher attack rate in HIV-positive subjects than in HIV-negative subjects [16].

To reduce the risk of cryptosporidiosis due to drinking water, the intervention keys are catchment protection, appropriate treatment (especially the installation of a filtration step), and secondary disinfection, such as by ultraviolet light [17]. In Italy, especially in mountain areas, small drinking water plants that often distribute surface water after filtration and chlorination treatments or UV treatment serve many small villages. Moreover, in these areas, breeding activities and wild animals are often present; therefore, surface waters are potentially at risk of contamination by *Cryptosporidium* spp. In this context, the real infection risk should consider the oocysts’ viability. Among inactivation treatments, UV has been shown to heavily damage DNA, and even if membranes and enzymes seem to remain intact, the organism is no longer capable of replicating [18].

Both in vitro and in vivo methods have been developed to evaluate the viability and infectivity of oocysts. In vitro methods include parasite excystation, and fluorogenic dye inclusion/exclusion (4,6-diamidino-2-phenylindole, DAPI), coupled or not with DNA amplification. Moreover, the ability of *Cryptosporidium* to invade cells and replicate within them, such as the presence of specific mRNA monitored with reverse transcriptase-polymerase chain reaction (RT-PCR), could be used to evaluate the oocysts’ viability. In vivo methods consist of animal infectivity assays [19].

The aim of this study was to evaluate *Cryptosporidium* contamination using common microscopic and molecular methods in a drinking water plant located in a rural area of Italy in order to monitor the effectiveness of the UV treatment installed upstream and thereby reduce the risk for the population.

## 2. Materials and Methods

### 2.1. Sampling

The sampling was carried out in two small towns located approximately 600 m above sea level in a pre-mountainous area in northwest Italy. This area is rich in forests and wildlife. The drinking water is derived from a surface water resource (stream).

The environmental sampling was carried out for a period of three years, between May 2013 and May 2016, and three different points were monitored: the drinking water source (river, n = 6, IN), the effluent of the drinking water treatment plant (n = 9, OUT), and tap water (n = 9) from a public drinking fountain located in a district of the municipality selected as a collection point along the network. The water samples were filtered through Envirochek HV capsules (Pall Corporation, VWR International Srl, Milan, Italy) with a flow rate of 2 L/min. The number of liters sampled varied from 155 to 1500, related to the water characteristics. The capsules were refrigerated at 4 °C until sample processing. All samples were processed within 24 hours of sampling.

### 2.2. Drinking Water Treatment Plant

The municipality is equipped with a drinking water treatment plant (DWTP, with a productivity ranging from 14 to 35 m^3^/h) which consists of the following phases: water catchment from the stream, peroxidation with sodium hypochlorite (approximately 1.5 mg/L), filtration on sand, filtration on Granular Activated Carbon (GAC), final chlorination (0.15–0.20 mg/L of residual chlorine) and UV disinfection (minimum applied dose 400 J/m^2^), performed with a Wallace and Tiernan Barrier® M900 UV system equipped with medium pressure lamps, designed for the treatment of 10–1150 m^3^/h of water flow.

### 2.3. Cryptosporidium Detection by IFA/DAPI

The presence of *Cryptosporidium* oocysts was assessed according to the Italian Official Methods for water samples [20]. Briefly, oocysts were eluted by twice washing the capsules with 125 mL of eluting solution on a laboratory shaker at 600 osc/min for 5 min. Then, the eluted samples were concentrated to a 10 mL volume by centrifugation at 3500 rpm for 10 min and submitted to Immunomagnetic Separation (IMS), as recommended by the manufacturers (Dynabeads GC-Combo, Invitrogen Dynal, A. S., Oslo, Norway). At this step, an aliquot of a sample was spiked with ≈1000 viable oocysts (1–6 months of age) suspended in PBS and supplemented with antibiotics provided by Waterborne, Inc. (New Orleans, LA, USA) to evaluate recovery of the IMS (positive control). Of the 110 µL obtained by IMS, 55 µL were used for an immunofluorescence test using the fluorescently labelled monoclonal antibody *Cryptosporidium* Cell Test IF (Cellabs, Sidney, Australia). Slides were examined using an epifluorescence microscope. Oocysts showing typical, confirmatory features (ovoid or spherical shape, size 4–6 µm, brilliant apple-green fluorescence) were enumerated, and the numbers referred to the concentrations of parasite oocysts per 100 L of water (reference volume for parasitic protozoa according to Italian law). The mean recovery of the elution and concentration phases is 30% (± 5%), as reported in the Italian Official Method [20]. The mean recovery of the IMS phase was 99% (± 3%), and it was evaluated with the following formula:

Recovery% = 100 × [(Nsp − Nusp)/T]
  Nsp = number of oocysts counted in the spiked sample
  Nusp = number of oocysts counted in the unspiked sample
  T = true value of the oocysts or cysts spiked
The oocyst concentration was calculated with the formula reported below:
  N oocysts/100 L = [(Nm × 2)/Ls] × 100
  Nm = number of oocysts determined by microscopy
  Ls = number of liters sampled

The presence of nuclei in the oocysts (indicator of oocyst viability) was confirmed by staining with the nuclear fluorochrome 4’,6-diamidino-2-phenylindole (DAPI) (Sigma-Aldrich Srl, Milan Italy). The percentage of viability was calculated with the formula reported below:

Viability % = (Nn × 100)/No
Nn = number of oocysts with DAPI-positive nuclei
No = number of IFA-positive oocysts

### 2.4. Cryptosporidium Detection by Reverse Transcriptase-PCR (RT-PCR)

The remaining 55 µL of IMS-purified oocysts was used for RT-PCR. Briefly, the suspension was centrifuged; the pellet was resuspended in InstaGene Matrix (Bio-Rad, Segrate, Milano, Italy) and incubated at 45 °C for 20 min to maximize heat shock protein 70 (hps70) mRNA production. After heat shock, lysis-binding buffer (200 µL) provided in the kit (Dynabeads mRNA DIRECT kit, Thermo Fisher Scientific, Milan Italy) was added to each sample, and this mixture was then subjected to freeze-thaw treatment (five cycles in liquid nitrogen for 30 s and thawing at 37 °C) to disrupt oocysts and release nucleic acids. The debris was pelleted and the supernatant containing the mRNA was added to oligo(dT)25 magnetic beads. Extraction and isolation of mRNA were carried out according to the manufacturer’s instructions. RT-PCR was performed with the GeneAmp Gold RNA PCR Kit (Applied Biosystems, Thermo Fisher Scientific, Milan, Italy). Details about the RT-PCR protocol for the detection of the *C. parvum* and *C. hominis* hsp 70 gene were previously developed [21]. The presence of the specific band corresponding to *C. parvum* and *C. hominis* (590 pb) was assessed by comparison with the amplicon obtained from spiked samples (positive control). A t-test was used to compare the mean oocyst concentration and was performed by the statistical package SPSS 25.0 (SPSS for Windows, Chicago, IL, USA).

### 2.5. Cryptosporidium spp. PCR

For species identification, DNA was extracted from IMS-captured oocysts by using the FastPrep120 apparatus and the FastDNA kit (MP Biomedicals, Santa Ana, USA). A nested PCR assay was used to amplify a ~590 bp fragment of the small subunit rRNA (SSU rRNA) gene, as previously described [22]. PCR conditions, for both primary and secondary amplification, were as follows: after an initial denaturation step at 94 °C for 3 min, 40 cycles of denaturation at 94 °C for 30 sec, annealing at 50 °C for 1 min and extension at 72 °C for 1 min were performed, followed by a final extension step at 72 °C for 7 min. Negative (no template) and positive (DNA from the *C. parvum* Moredun strain) controls were included in each experiment. PCR was performed using 25 µL of 2X GoTaqGreen (Promega, Madison, USA), 10 pmol of each primer, 5.0 µL of DNA, and nuclease-free water up to a final volume of 50 µL. Reactions were performed on a Perkin Elmer 9700 apparatus (Life Technologies, Carlsbad, USA). Aliquots of PCR products (10 µL) were loaded on a 1.5% agarose gel stained with ethidium bromide. PCR products were purified using spin columns (QiaQuick PCR purification kit, Qiagen, Milan, Italy), and sequenced on both strands. Sequences were edited and assembled using the software package SeqMan 7.1 (DNASTAR, Madison, USA). BLASTn searches (http://blast.ncbi.nlm.nih.gov/Blast.cgi) against the GenBank database were used to identify *Cryptosporidium* at the species level.

## 3. Results and Discussion

The concentrations of *Cryptosporidium* spp. in the different water samples analyzed are reported in Table 1.

Oocysts were found in most samples (83.3%), at both the influent (IN, 100% positive) and effluent (OUT, 77.8% positive) of the DWTP, as well as at the designated collection point along the network (tap water, 77.8% positive), with the exception of samples collected in May and June 2013. In the sampling carried out in August 2013, the influent and effluent of the DWTP were characterized by a low contamination level. Conversely, a relatively higher contamination of tap water was observed. Although samples were collected simultaneously and thus a real relation between the influent, effluent and tap water was not possible, we can speculate that the high contamination in the tap water sample could be related to an instantaneous peak of oocysts in the raw water, only partially or not at all removed by the potabilization treatment.

The results obtained highlighted a high variability of oocyst concentrations in all samples analyzed (mean 4.4 ± 5.8 /100 L). As reported in Table 1, few peaks of contamination occurred in the months of August and October 2013, and in May 2016, in both raw and treated waters, and this could be attributed, at least in part, to the increased water flow due to snow melting or increased rainfall, which normally occur during these periods.

**Figure 1:**
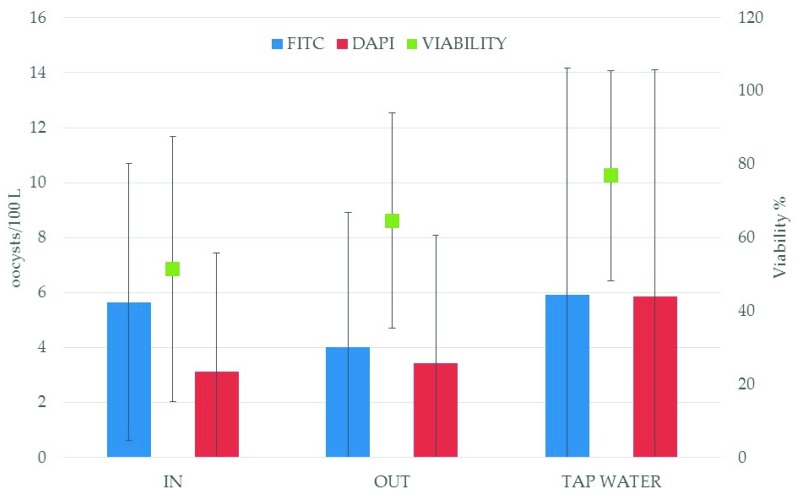
Mean and standard deviation (SD) of the concentration of *Cryptosporidium* spp. and number of oocysts viable at the different sampling points. The percentage of viable oocysts in the sample was calculated using DAPI staining.

The mean values and the standard deviations of the oocyst concentrations calculated in the 3 sampling points are shown in Figure 1. The mean values of the effluent were slightly lower than the influent values, indicating that the DWTP partially retained the oocysts, even though the difference was not statistically significant (*t*-test).

A high variability was also observed in the percentage of samples showing the presence of intact nuclei as determined by DAPI staining (all samples mean, 68.1% ± 29.9%). In the samples analyzed at the effluent of the DWTP, the percentage of viable oocysts was similar to that of the influent (Figure 1), suggesting that the high-power UV treatment, inserted in this system to inactivate the oocysts, did not seem to produce the desired results. The UV treatment was installed in this DWTP to reduce the risk of microbial contamination of the drinking water distributed to the population, because the surface water source was derived from a mountain creek. This water source is characterized by a variable flow rate depending on the season and the amount of rainfall. Therefore, the concentration of microorganisms entering the DWTP can vary greatly.

It is important to highlight that exposure to UV could cause irreversible damage to *Cryptosporidium* oocysts’ DNA, causing their inactivation [18], although some studies have shown an influence of water turbidity on the oocyst inactivation [23].

The process of UV inactivation and damage is not immediately observable, so it can be expected to find apparently viable oocysts in the samples; moreover, DNA is quite resistant and may be present in non-viable or irreparably damaged cells. In these cases, the DAPI dye penetrates into the cell, linking to the genetic material and giving rise to false positives [24,25].

Considering the limitations of DAPI staining and the importance that the viability of pathogens has in public health concern, an evaluation of oocyst viability was carried out by RT-PCR. Interestingly, none of the samples analyzed was positive according to RT-PCR.

The microscopic analysis method employed does not allow the identification of *Cryptosporidium* at the species level, since during immunomagnetic separation (IMS), common antibodies for all *Cryptosporidium* species are used to link microspheres and oocysts, and the direct immunofluorescence approach was based on an antigen binding an antibody common to the whole genus.

In contrast, RT-PCR was performed to specifically assess the viability of *C. parvum* and *C. hominis*. To clarify the results obtained, a nested PCR targeting the 18S ribosomal DNA gene was performed to identify *Cryptosporidium* species on samples collected in July 2015 (IN DWTP and Tap Water). The PCR products obtained were sequenced and compared with public databases (BLAST search system). The nucleotide sequences determined in this study were deposited in the GenBank database under the accession numbers MK402221 and MK402222. The results confirmed the presence of *Cryptosporidium* spp. in both samples. The comparison indicated that the sequence found in the IN of the DWTP sample was very closely related to a genotype found in bank voles (99% with GenBank accession numbers KY644594 and KY644595). The sequence found in the tap water sample showed a high similarity (97%) with a sequence found in a drinking water sample in Scotland (GenBank accession number HM015878). These data, coupled with the negative results obtained using the RT-PCR method developed to evaluate the viability of the common human pathogens *C. parvum* and *C. hominis*, suggest that the waters examined were contaminated by feces from wild rodents.

The method used during this study (including the RT-PCR protocol) is the Italian Official Method that arose from the EPA (Environmental Protection Agency) method 1623 [26]. This is the most widely used method worldwide, but it is still expensive and complex. Moreover, it requires much experience, especially in microscopic determination. For this reason, the search for *Cryptosporidium* oocysts is not an analysis that is routinely performed by the Italian laboratory of the DWTPs, but only if the drinking water comes from surface waters that can present problems of contamination by these protozoa.

The vulnerability of surface water with respect to *Cryptosporidium* contamination is confirmed in the literature. It also emerges that *C. parvum* and *C. hominis* remain the species that are mostly found both in surface waters and in treated waters worldwide.

In a recent study conducted in Minnesota, it was found that 40% of the 145 wells examined were contaminated with *Cryptosporidium* spp.; furthermore, out of 45 samples sequenced, 41 were positive for *C. parvum*, 2 were positive for *C. hominis*, and 2 were positive for *C. andersoni* [27]. Following the examination of the 178 outbreaks that occurred in England and Wales between 2009 and 2017, it was highlighted that the transmission of the protozoan occurred mainly via recreational water, animal contact, environmental contact, person-to-person spread, drinking water and food. Furthermore, *C. parvum* was identified in 69 outbreaks and *C. hominis* in 60 cases, while in two cases, both species were identified. In one of the two episodes caused by drinking water, *C. hominis* IbA10G2, *C. parvum* and *C. andersoni* were identified in the source water, and *C. hominis* in treated water. *C. hominis* IbA10G2 and IdA18 were identified in the cases [28]. During a study conducted in Colombia, the authors investigated the presence of *Cryptosporidium* spp. in both raw and treated water samples used for the production of drinking water. *Cryptosporidium* spp. were only detected in raw waters both in urban areas and in rural areas. In the *Cryptosporidium*-positive samples, the authors identified *C. parvum* (Subtype IIcA5G3c), *C. galli*, *C. molnari*, *Cryptosporidium* sp. genotype II of bats and *Cryptosporidium* sp. genotype VIII of birds [29]. The spreading of the *Cryptosporidium* genotype associated with wild animals was also confirmed in our results. Only in Colombia were species found mainly circulating in animals, even if they were different from those species found in the samples of this study. It is also important to remember that in June 2008 in England, an outbreak was caused by *Cryptosporidium* spp., which resulted in 422 cases, of which 32 cases were ascertained with clinical analyses. The concentration of oocysts ranged from 0.0005 to 0.08 oocysts/L in drinking water. Molecular analyses have shown that *C. cuniculus*, a typical species of rats, is also capable of infecting humans. The problem was caused by a fresh wild rabbit carcass found below the inlet pipe to a backwash granulated activated carbon tank [30].

## 4. Conclusions

The results show the presence of *Cryptosporidium* spp. in the water source analyzed. Water sampled at the outlet of the drinking water treatment plant was usually contaminated by *Cryptosporidium* spp., even if the average oocyst concentration was lower than that in the supply water. The presence of the protozoa in the outlet of the DWTP can be attributed both to the lack of retention of the protozoa by the filtration phase and to the resistance of the oocysts to standard disinfectants, such as chlorine.

The results obtained using the molecular protocol (RT-PCR) highlighted that this method can be useful to evaluate the viability of the oocysts of *C. parvum* and *C. hominis*, which are the most important species for human health. Further analysis carried out by nested PCR on the samples of July 2015 revealed the presence of genotypes likely derived from wild animals (i.e., bank vole), which should not represent a risk for human health. In this rural and pre-mountainous area, there are no cattle and sheep farms, so wild animals and birds can contribute more to surface water contamination. Although no contamination by pathogenic *Cryptosporidium* species was detected in the water samples investigated, the results obtained show that this stream can be classified as a source of risk and indicate the need for continuous control of this water resource. The supply source and the drinking water treatment plant examined are a classic example of a system that requires continuous monitoring to guarantee population health. It is possible that a similar situation exists in many areas near the Italian Alps or Apennines. Therefore, this experience should motivate the control of water resources for human consumption in these areas by competent authorities. Furthermore, the effectiveness of the UV treatment plant must be constantly monitored and possibly modified, making it more effective at inactivating protozoa in order to provide additional protection for the user population. This finding, in turn, underlines the importance of also monitoring unconventional (non-bacterial) biological contaminations (protozoa) in rural areas, including those of developed countries, thereby enhancing the importance of tailored water safety plans.

## Figures and Tables

**Table 1:** Details of sampling; concentrations and viability of *Cryptosporidium* spp. in the different water samples analyzed.

Date	Sampling Point	Liters Filtered	FITC Oocysts/100 L ^a^	DAPI Oocysts/100 L ^b^	Viability % ^c^
May, 2013	OUT DWTP	352	ND	ND	NC
	Tap Water	568	ND	ND	NC
June, 2013	OUT DWTP	480	ND	ND	NC
	Tap Water	742	ND	ND	NC
August, 2013	IN DWTP	303	1.98	ND	NC
	OUT DWTP	294	0.68	ND	NC
	Tap Water	372	20.43	19.89	97.36
October, 2013	IN DWTP	252	12.7	3.17	24.96
	OUT DWTP	375	13.87	11.74	84.64
	Tap Water	402	1.49	0.50	33.39
December, 2013	OUT DWTP	300	2.00	0.67	33.50
	Tap Water	375	1.07	ND	NC
March, 2014	IN DWTP	268	4.48	2.24	50.00
	OUT DWTP	391	1.53	0.51	33.33
	Tap Water	455	1.76	0.88	50.00
April, 2014	IN DWTP	296	2.03	0.68	33.50
	OUT DWTP	324	2.47	1.23	49.80
	Tap Water	584	1.37	1.37	100.00
July, 2015	IN DWTP	413	1.45	1.45	100.00
	OUT DWTP	382	0.26	0.26	100.00
	Tap Water	1,500	0.33	0.33	100.00
May, 2016	IN DWTP	155	11.29	11.29	100.00
	OUT DWTP	610	7.21	6.23	86.41
	Tap Water	810	15.06	12.10	80.34

ND: not detected; NC: not calculated; ^a^ oocysts stained with fluorescently labelled monoclonal antibody for *Cryptosporidium* spp. (FITC); ^b^ oocysts that presented nuclei stained with DAPI; ^c^ percentage of viable oocysts in the sample calculated using DAPI staining.
